# Self-Control in Aiming Supports Coping With Psychological Pressure in Soccer Penalty Kicks

**DOI:** 10.3389/fpsyg.2019.01438

**Published:** 2019-06-27

**Authors:** José A. Navia, John van der Kamp, Carlos Avilés, Jesús Aceituno

**Affiliations:** ^1^Facultad de Ciencias de la Actividad Física y del Deporte, Departamento de Ciencias Sociales, de la Actividad Física y del Ocio, Universidad Politécnica de Madrid, Madrid, Spain; ^2^Faculty of Behavioral and Movement Sciences, Department of Human Movement Sciences, Vrije Universiteit Amsterdam, Amsterdam, Netherlands; ^3^Research Centre for Exercise, School and Sport, Windesheim University of Applied Sciences, Zwolle, Netherlands; ^4^Facultad de Educación, Departamento de Didáctica de las Lenguas, Artes y Educación Física, Universidad Complutense de Madrid, Madrid, Spain

**Keywords:** football (soccer), autonomy, anxiety, performance, penalties

## Abstract

This study addressed the question whether coaches better allow athletes to self-control their decisions when under pressure or whether to impose a decision upon them. To this end, an experiment was conducted that manipulated the soccer kickers’ degree of control in decision-making. Two groups of elite under-19 soccer players (*n* = 18) took penalty kicks in a self-controlled (i.e., kickers themselves decided to which side to direct the ball) and an externally controlled condition (i.e., the decision to which side to direct the ball was imposed upon the kickers). One group performed the penalty kick under psychological pressure (i.e., the present coaching staff assessed their performance), while the second group performed without pressure. Just before and after performing the kicks, CSAI-2 was used to measure cognitive and somatic anxiety and self-confidence. Further, the number of goals scored, ball placement and speed, and the duration of preparatory and performatory behaviors were determined. The results verified increased levels of cognitive and somatic anxiety after performing the kicks in the pressured group compared to the no-pressure group. In addition, degree of self-control affected the participants’ performance, particularly in the pressured group. They scored more goals and placed the kicks higher in the self-controlled than in the externally-controlled condition. Participants also took more time preparing and performing the run-up in the self-controlled condition. Findings indicate that increased self-control helps coping with the debilitating effects of pressure and can counter performance deteriorations. The findings are discussed within the framework of self-control theories, and recommendations for practitioners and athletes are made.

## Introduction

In the last decade or so, and particularly with the introduction of mobile gaze-trackers, soccer penalty kicking has attracted a growing interest among researchers. The soccer penalty kick provides a well-defined situation, granting researchers to study the (isolated) effects of constraints manipulation on interpersonal interactions in well-controlled (competitive) situations. At the same time, soccer penalty kicks, for example by winning or losing penalty shoot-outs in international soccer tournaments, have a tremendous socio-economic impact.

The probability of scoring a penalty kick is approximately 80% (Palacios-Huerta, 2003). This high success rate basically reflects the spatio-temporal demands of the situation; typically, a goalkeeper has less time available than they require to dive and intercept the ball (Van der Kamp et al., 2018). In other words, the advantage is clearly for the kicker (and should be, given that it was introduced as a punishment). The advantage for the penalty kicker comes with high expectancies of success. Such high expectancies can provoke adverse psychological effects, such as increased pressure and enhanced anxiety levels, which in turn may hamper performance. Psychological pressure is further increased when players are being evaluated or socially judged (reviews on Hill et al., 2010; Mesagno and Beckmann, 2017). Accordingly, when there is much at stake, and pressure is high, penalty takers’ performance is negatively affected (Dohmen, 2008; Arrondel et al., 2019).

In addition, previous work has shown that soccer penalty kickers under pressure may behave in ways that can hinder performance maximization. Under pressure, players are more inclined to rush the preparation and the execution of the run-up toward the ball. Players who do show these avoidance coping behaviors converted 20% fewer penalties than those who take their time (Jordet et al., 2009). Research also shows that psychological pressure directly affects players’ aiming. For example, in competition, penalty kicks are shot over four times more frequently toward lower zones of the goal than to the upper zones, despite the probability of scoring being significantly higher when the ball is directed toward upper areas of the goal (Bar-Eli and Azar, 2009; Almeida et al., 2016). Players seem to prefer the risk of a (low) ball being stopped by the goalkeeper over the risk of directing the (high) ball over the goal. Possibly, with respect to the latter failure, players may be apprehensive to be perceived as unskilled (Bar-Eli et al., 2009; Bar-Eli and Azar, 2009). In addition, experimental laboratory studies have indicated that increased anxiety negatively impacts attentional control and the aiming of the kick. Specifically, penalty takers looked longer toward the goalkeeper (and less toward the aiming location and/or the ball) and placed the penalties closer to the goalkeeper under high anxiety conditions (Wilson et al., 2009; c.f., Noël and Van der Kamp, 2012).

One influential account for explaining how athletes successfully cope to achieve their goals under pressure focusses on *perceived control* of the task, which refers to the athlete’s beliefs about the degree of control they have over the task (Hanton et al., 2003; Jordet et al., 2006). Perceived control is conceptualized to consist of contingency expectations and self-competence. Contingency relates to the expectations of relationship between the athlete’s action and the outcome, whereas self-competence perceptions relate the beliefs about the own ability (Skinner, 1996). Evidence suggests that increased anxiety can actually have a facilitative effect on performance provided the athletes maintain a good level of confidence of competence (Hanton and Connaughton, 2002; Hanton et al., 2003). Accordingly, with respect to soccer penalty kicks, Jordet et al. (2006) carried out retrospective interviews among elite players, who participated in a shoot-out during the European Championship for nation teams. They reported that penalty takers with relatively low perceived competence and contingency (i.e., players who attribute performance success to luck rather than skill) experienced enhanced cognitive anxiety and/or interpreted their somatic anxiety as more debilitative to their performance compared to players with high perceived competence and contingency. More recently, Wood and Wilson (2012) found that penalty kickers who followed a perceptual training (i.e., directing visual attention toward the aiming location prior to the kick) not only improved gaze but also reported enhanced perceived control of the task both with regard to contingency and self-competence. The enhanced perceived control was accompanied by an increment in the players’ ability to cope with the pressure and to score. Yet, while these works show that perceived control is associated with the ability to cope with psychological pressure during penalty kicks (Jordet et al., 2006; Wood and Wilson, 2012; Wood et al., 2015), it has remained unclear to what extent the purported advantages of those (often retrospective) perceptions are underpinned by *actual control* of the task (rather than perceived control). Actual control is defined as the extent to which an individual can intentionally produce a desired outcome. Although actual control and perceived control are typically related, changes in actual control does entail modifications in objective control of context conditions and/or persons interactions or actions, unlike perceived control which might just be illusionary (Skinner, 1996).

One modality within actual control is an individual’s autonomy in the control of and/or making decisions about intended actions, that is, the degree to which the individual can modulate the means that define task control. For example, self-controlled and externally controlled actions differ in the degree to which an athlete can choose (or not) pertinent aspects of task execution when trying to achieve the task goal. Typically, increasing self-control (e.g., having players decide upon amount of practice, the order of different practice trials, the use of models for observation, the use of assistive devices, or provision of feedback) enhances motor performance and/or learning (for overview, Wulf, 2007). For instance, in an early study by Janelle et al. (1997), which originated this line of research, participants showed better retention in the far-aiming task of ball throwing when they allowed to choose when to use evaluative video-feedback during practice in comparison to yoked counterparts who were not free to choose when to use feedback. Yet, these effects of self-control have not been thorougly investigated in elite players under condition of psychological pressure.

In soccer penalty kicks, Scurr and Hall (2009) manipulated the autonomy of players regarding the choice of the angle of approach during the run-up of kicks in no-pressure situations. In this case, the non-professional, intermediately skilled players showed similar kicking accuracy irrespective of whether they self-controlled the approach angle or whether it was pre-scribed or externally controlled. Other experimental studies in penalty kicking have used both self-controlled and externally controlled instructions for choosing the side and/or height for the placement of the ball (Morris and Burwitz, 1989; Furley et al., 2012), often in interaction with other penalty kick strategies (Castillo et al., 2010). Yet, these studies did not systematically manipulate and compare the efficacy of self- and externally controlled kicks.

In sum, the evidence indicates that with increased psychological pressure penalty kick performance deteriorates, among others because of poor decision-making about ball placement. It may therefore be anticipated that players would improve performance when they would follow instructions that would result in a more rational distribution of ball placement. However, the associated reduction of self-control over the task may adversely affect coping in pressure situations, which in turn would deteriorate performance. This has not been directly investigated. Hence, the present study manipulated the degree of autonomy of soccer penalty takers in choosing the aiming location (i.e., side and height). The players took penalty kicks in a self-controlled condition (i.e., they decided about the aiming location themselves) and in an externally controlled condition (i.e., the aiming location was imposed upon them). One group performed the kicks in a pressured condition and the second group in a no-pressure condition. It was predicted that self-control would lead to superior performance (i.e., goal scoring, ball placement and speed) and execution (i.e., preparatory and execution durations), in particular in the pressured group.

## Materials and Methods

### Participants

Eighteen elite under-19 soccer players (*M* = 17.72 years old, SD = 0.83) volunteered to participate. Participants were all playing for a club that competes in the highest league in Spain for under-19 players (i.e., playing experience: *M* = 12.28 years, SD = 1.45). In addition, four goalkeepers, who were matched to the penalty takers in age (*M* = 17.40 years old, SD = 1.14), level (same league), and experience (*M* = 10.80 years, SD = 1.6) were recruited to act as actual goaltenders. Ethical approval was obtained from the local University’s ethics committee, and all players provided written consent prior to the start of the experiment.

### Equipment

**Figure 1:**
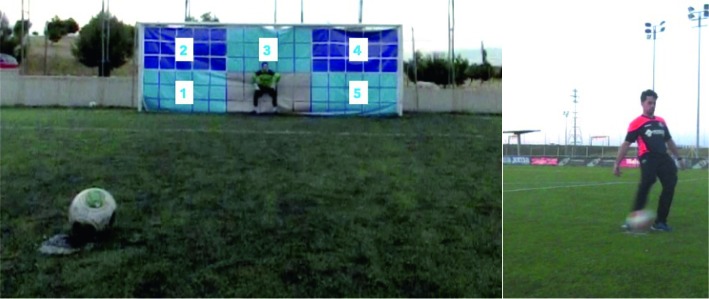
Set up of the experiment depicting the perspective of the goal (left) and the penalty takers (right).

The experiment was conducted on an outdoor artificial turf pitch. Dimensions of the goal (7.32 m × 2.44 m), ball type (size 5), and distance to the penalty mark (11 m) were in accordance with FIFA laws (IFAB, 2016). The goalmouth was fully covered with a canvas, which was divided in six equal areas of 2.44 m × 1.22 m (Figure 1, left). Each area was subdivided in 15 zones of 0.48 m × 0.40 m. Two cameras were employed to record the penalties. The first camera (Casio Exilim FH 100, Japan, 120 fps mode) was placed 2 m to the side and 1 m behind the penalty spot (Figure 1, left), in such way that recording perspective captured both the penalty taker’s football contact and the outcome of the kick (i.e., the moment and location of the ball landing in the goal). A second camera (Casio Exilim FH 100, Japan, 30 fps mode) placed 3 m to the side and 2 m in front of the penalty spot recorded the preparatory actions of penalty takers (Figure 1, right). All recordings were analyzed with Kinovea V.0.8.25.

Finally, the anxiety of the players was assessed by means of the Competitive State Anxiety Inventory-2 (CSAI-2; Martens et al., 1990) in its revised Spanish version (CSAI-2R; Andrade et al., 2007). The questionnaire consists of 16 items (Likert-type scale from 1 to 4) distributed in three dimensions: (1) cognitive anxiety (five items), typified by negative self-images and self-doubts; (2) somatic anxiety (six items), which refers to physiological responses (e.g., increased heart rate, tense muscles, and clammy hands); and (3) self-confidence (five items) regarding the positive expectations of success (Andrade et al., 2007).

### Procedure and Design

Data collection was carried out at the players’ habitual training venue. Each participant received the same general instructions about the experimental session beforehand in the dressing room. They were, however, not told about the experimental conditions and/or the exact aims of the experiment. After having received the general instructions, they completed the CSAI-2R. After a self-selected warm up (i.e., lasting approximately 10 min), the participants performed four familiarization trials. Then, each participant took 10 experimental penalty kicks, and then, immediately afterward, completed the CSAI-2R once again.

The 10 experimental kicks were divided in five self-controlled and five externally controlled kicks. The players were encouraged to score as many of the 10 kicks as possible, whereas goalkeepers were told to do their very best to save them. In the self-controlled condition, the penalty takers were instructed to freely choose the area of the goal to which to aim the ball. In the externally controlled condition, the experimenter instructed the players before the start of the run-up about the area of the goal where they had to place the ball, one shot toward each of the five outer areas (i.e., areas 1 to 5, Figure 1, left). The order of self-controlled and externally directed kicks was randomized using the Excel’s “random” algorithm. In order to prevent that the goalkeeper became of aware of the two conditions, penalty takers followed the same routine regardless the condition. Players approached to researcher to take the ball from outside the penalty box and were shown that the goal area could be freely chosen or to which area to aim. By instructing them to pick a goal area before the start of the run-up, the procedure in the self-controlled condition promoted players to use the keeper-independent strategy (Van der Kamp, 2006; Noël et al., 2014). However, players were not explicitly instructed about it to prevent ironic effects (Bakker et al., 2006). In both conditions, there were no additional instructions regarding deception (Dicks et al., 2010) or run-up length (Van der Kamp, 2006), or angle of approach (Scurr and Hall, 2009). Finally, the players placed the ball on the penalty spot, prepared for the run-up, and executed the run-up to the ball and the kick.

Participants were ranked on the basis of the individual ability on penalty kicks and assigned to the pressure and no-pressure groups such that the two groups matched in ranking. To this end, information of the individual ability of the players was provided by the technical staff (i.e., coach and/or trainers) based on players’ performance records, skill level, and previous involvement experience on competitive penalty kicks (Plessner et al., 2009). The pressure group performed the penalty kicks in the presence of the head coach and two members of the technical staff (i.e., assistant coach and physical trainer who took written notes of their performance) and were told that further decision regarding upcoming penalty shout outs of the team would be based on their performance (Note: players were fully debriefed after the experiment). By contrast, the no-pressure group took the penalty kicks only in the presence of the goalkeeper and researcher, and no further information on the importance of their performance was given.

### Data Analysis

To verify anxiety, the cognitive, somatic, and self-confidence scores on the CSAI-2R were used. Performance was assessed offline from the video recordings. First, the number of penalties scored was counted. Second, the location of the kicks was determined by the area of the goal (i.e., at the center of the 15 smaller rectangles of the six areas) the ball contacted the canvas (or was intercepted) and calculated in vertical distance from the ground (i.e., height of the kick in cm) and horizontal distance from the goal center (i.e., extent of the kick in cm). Finally, the speed of the kick was calculated based on ball-flight time (i.e., differences between the moment of foot-ball contact and the moment the ball contacted the canvas or was intercepted) and the distance the ball travelled.

Two measures were used to calculate the duration of the preparatory and execution phase of the penalty kick (Jordet et al., 2009; Furley et al., 2012). The duration of the preparation phase was defined as the time between the placement of the ball on the penalty spot and the start of the run-up. The duration of the execution phase was defined as the time from the start of the run-up toward the ball until the football contact.

### Statistics

To verify the effectiveness of the pressure manipulation, the ratings for anxiety were submitted to Mann-Whitney test to compare groups, whereas Wilcoxon tests were performed to compare differences between the two assessments before the experiment and immediately after. Effect sizes were expressed in *r*, with 0.1, 0.3, and 0.5 for small, medium, and large effects, respectively (Cohen, 1988). The remaining dependent variables were submitted to separate 2 (group: pressure vs. no-pressure) by 2 (autonomy: self-controlled vs. externally-controlled) Mixed Design Analysis of Variance (ANOVA). *Post hoc* comparisons were performed with t-test and Bonferroni corrections. Partial effect sizes of ANOVA’s were expressed using ($\eta_{p}^{2}$), with values of 0.01, 0.06, and 0.14 representing small, medium, and large effects, respectively (Cohen, 1988). In case that the homogeneity and sphericity assumptions of the measures were not met, Mann-Whitney and Wilcoxon tests were conducted instead. IBM SPSS V. 24 was used to carry out statistical analysis.

## Results

### Anxiety Measures

**Figure 2:**
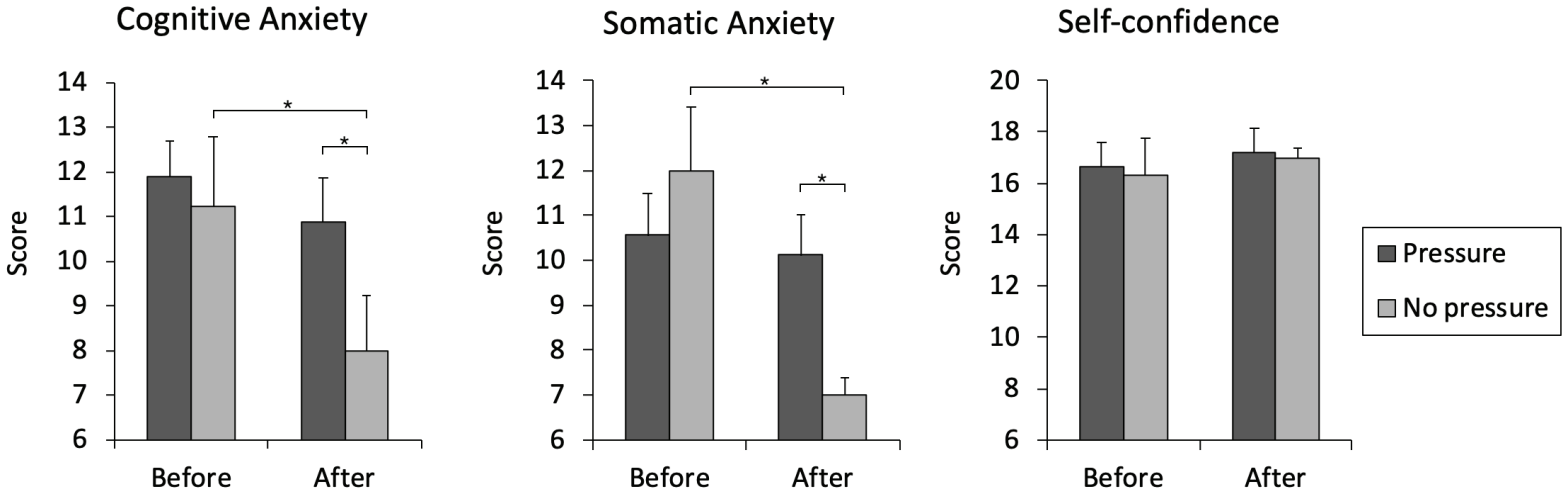
Scores of CSAI-2R of the players before and after performing the penalty kicks. *p < 0.05.

Figure 2 suggests that anxiety levels were similar between groups before taking the penalty kicks but lower for the no-pressure group directly after the experiment. In other words, whereas participants of the pressure group reported moderately to high levels of anxiety throughout the experiment, these levels showed a steep drop for the no-pressure group. Accordingly, just before the experiment, no differences were found between groups for cognitive anxiety (*U* = 39.50, *p* = 0.465, *r* = −0.02), somatic anxiety (*U* = 27.50, *p* = 0.129, *r* = −0.27), or self-confidence (*U* = 35.00, *p* = 0.333, *r* = −0.12). However, immediately after the experiment, the no-pressure group showed significant lower levels of cognitive anxiety (*U* = 20.00, *p* = 0.038, *r* = −0.43), and somatic anxiety (*U* = 17.50, *p* = 0.020, *r* = −0.49) compared to pressure group (exact, unilateral). The self-confidence scores did not differ significantly between groups.

Furthermore, the no-pressure group significantly decreased both the cognitive anxiety (*z* = −1.97, *p* = 0.047, *r* = −0.66) and somatic anxiety (*z* = −2.67, *p* = 0.004, *r* = −0.89) during the experiment, whereas the pressure group maintained the scores over time (*p*’s > 0.40). Differences in self-confidence across time were not found (Figure 2).

### Performance Measures

**Figure 3:**
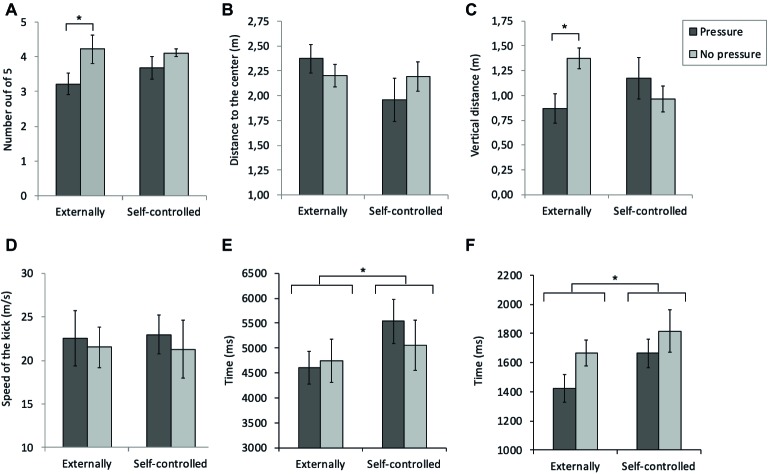
Performance measures of the kicks **(A–D)** and penalty takers behavior **(E,F)**. Error bars represent standard error of the mean. *p < 0.05.

Figure 3A shows that number of goals scored by the pressure group was lower than by the no-pressure group. Interestingly, it also suggests smaller differences between groups for the self-controlled compared to externally controlled condition. This was confirmed by Mann-Whitney test showing an effect of group on the number of goals scored only within the externally controlled condition (*U* = 19.00, *z* = −1.97, *p* = 0.042, *r* = −0.46) and not for the self-controlled condition (*U* = 33.00, *z* = −0.79, *p* = 0.543, *r* = −0.19). The remaining comparisons were not significant.

Autonomy had no effect on the horizontal extent (i.e., how far the ball was aimed from the goal center) as the mixed ANOVA did not reveal a significant main effect for autonomy, *F*(1,16) = 2.30, *p* = 0.149, $\eta_{p}^{2}$ = 0.13, and also not for group, *F*(1,16) = 0.031, *p* = 0.862, $\eta_{p}^{2}$ < 0.01, or their interaction, *F*(1,16) = 1.98, *p* = 0.178, $\eta_{p}^{2}$ = 0.11 (Figure 3B). With respect to vertical distance, the effects of autonomy and group seem to interact (Figure 3C). Accordingly, mixed ANOVA did not found an effect for autonomy, *F*(1,16) = 0.12, *p* = 0.74, $\eta_{p}^{2}$ = 01, and group, *F*(1,16) = 1.04, *p* = 0.323, $\eta_{p}^{2}$ = 0.06, but did reveal a significant interaction between autonomy and group (*F*(1,16) = 4.81, *p* = 0.043, $\eta_{p}^{2}$ = 0.23). *Post hoc* comparisons indicated that the pressure group placed the kicks lower than the no-pressure group but only in externally controlled condition (Figure 3C). In addition, we considered for the externally controlled condition to what degree the participants adhered to the instructions regarding the to-be-kicked height^1^. One-sample *t*-tests revealed that the pressure group aimed the kicks lower than instructed, *t*(8) = −3.18, *p* = 0.013, *df* = −0.47, *d* = −1.05, while no such difference with the instructed height was found for the no-pressure group, *t*(8) = 0.30, *p* = 0.773, *df* = 0.03, *d* = 0.11 (Figure 3C).

The speed of the kicks ranged between 17.9 and 28.2 m/s. Mixed ANOVA did not reveal significant effects of autonomy, *F*(1,16) = 0.026, *p* = 0.875, $\eta_{p}^{2}$ < 0.01, group, *F*(1,16) = 1.39, *p* = 0.256, $\eta_{p}^{2}$ = 0.08, or the interaction between the two factors, *F*(1,16) = 0.28, *p* = 603, $\eta_{p}^{2}$ = 0.02 (see Figure 3D).

Penalty takers took less time to prepare the kick in the externally controlled than during self-controlled condition, but this was not affected by pressure (Figure 3E). Accordingly, mixed ANOVA showed a main effect with a large effect size for autonomy on preparation time, *F*(1,16) = 7.76, *p* = 0.013, $\eta_{p}^{2}$ = 0.33, but no effects for group, *F*(1,16) = 0.08, *p* = 0.776, $\eta_{p}^{2}$ < 0.1, or between autonomy and group, *F*(1,16) = 1.87, *p* = 0.191, $\eta_{p}^{2}$ = 0.10) were found. Finally, Figure 3F illustrates the duration of the execution phase. Again, the duration for the externally controlled condition was shorter than in the self-controlled condition, *F*(1,16) = 11.64, *p* = 0.004, $\eta_{p}^{2}$ = 0.42. The mixed ANOVA did not reveal further effects of group, *F*(1,16) = 1.84, *p* = 0.194, $\eta_{p}^{2}$ = 0.10, or the interaction between the two factors, *F*(1,16) = 0.60, *p* = 0.451, $\eta_{p}^{2}$ = 0.04, on the run-up time.

## Discussion

This study examined the effect of actual self-control on coping with psychological pressure in a soccer penalty kick situation. To this end, we compared *in situ* performance of two groups of young, elite soccer players in conditions with different degrees of autonomy with respect to choosing the aiming location. Psychological pressure was effectively modulated by requiring them to take the penalty kicks in the presence of members of training staff, who–purportedly–would selectively evaluate their performances.

Research has shown that increased psychological pressure negatively affects the rate of scoring in penalty kicking (Dohmen, 2008; Wilson et al., 2009; Arrondel et al., 2019). In particular, increased pressure is associated with a hasted preparation and execution (Jordet et al., 2009) and sub-optimal ball placement (Bar-Eli and Azar, 2009; Almeida et al., 2016). The present study confirmed this: performances of the pressured group was worse compared to the no-pressure group. Importantly, however, this difference in performance was not found for the self-controlled condition. By contrast, in externally controlled condition, players under pressure actually showed signs of unsuccessful coping. They scored less goals and placed the kicks lower. Thus, the reduction in autonomy seems to be related to a reduction in the players’ ability to cope with anxiety, possibly resulting in the observed drop in performance.

This finding is in line with studies that examined individual self-control or autonomy in the performance and learning of motor skills. Typically, self-control in deciding when to obtain feedback or assistance has been shown to enhance learning and performance (e.g., Janelle et al., 1997). In our study, although participants were enticed to follow a goalkeeper-independent strategy (i.e., aim the ball toward an early chosen location, irrespective of the goalkeeper’s actions; Van der Kamp, 2006) in all instances, the reduction of autonomy in the externally controlled condition made the task constraints even more strict (i.e., the kicker was not allowed to change ball direction even if the goalkeeper would very clearly move in one direction long before the kick was completed). Consequently, penalty takers may have experienced a reduction of the control over the task during externally controlled kicks. The benefits of self-control have been explained by referring to motivational effects of increased autonomy, as indicated by concomitant increase in task interest, engagement, satisfaction, and self-efficacy (Sanli et al., 2013; Wulf and Lewthwaite, 2016). We did not gauge the motivational beliefs of the players in detail. Yet, we did find that the current participants’ ratings of self-confidence remained high across the entire experiment and equally so in both groups. It stands to reason, therefore, that the observed benefits of self-control or autonomy in coping with pressure should also be attributed to an increase in *actual* control on task performance –and not exclusively– to a change in perceived control or other motivational beliefs. Although we meant to elicit a goalkeeper-independent strategy (see above), it cannot be ruled out that sometimes (and more often under pressure) they switched to a goalkeeper-dependent strategy. This might (partly) explain the observed advantage in self-controlled condition in the pressured group. If so, that would be contrary to previous experimental work that showed a clear advantage of GK-independent strategy (Van der Kamp, 2006; Noël and Van der Kamp, 2012; c.f., Noël et al., 2014), or at least it raises further questions on how autonomy affects the degree of adherence to either a goalkeeper independent or dependent strategy. For instance, a kicker may intend to aim to a beforehand chosen area (i.e., independent strategy) unless she/he recognizes that the goalkeeper clearly moves in advance (e.g. longer than 600 ms before ball contact, Van der Kamp, 2011) toward that intended location.

The results also showed that durations of the different phases in the penalty kick sequence was affected to a greater extent by the degree of autonomy than by psychological pressure. The pressure group did not show a significant hastening behavior (c.f., Jordet et al., 2009), but they did significantly reduce the preparation and run-up times in the externally controlled compared to the self-controlled condition. For the preparatory phase, this may be due to absent and/or reduced decision-making and planning behaviors in the externally controlled condition. During run-up phase, the slightly extra time might suggest adaptive behaviors to the actual situation, such as, responding to the goalkeeper (see above).

For future work, we recommend to include more qualitative measures (e.g., extra measures as perceived control or contingency, but also including confrontation interviews; Jordet et al., 2006) to find out in more detail what players experience in in the different conditions. For instance, whether the anxiety levels are linked to the degree of autonomy control, or how the players experience cope with pressure during the performance of the penalty kicks. In this sense, note that anxiety levels of the penalty takers were already high in the period that preceded the kicks. The group exposed to pressure maintained those levels moderately high, whereas perceived anxiety of the no-pressure group dropped. This particular fluctuation of anxiety is consistent with previous observations among expert penalty takers (Jordet and Elferink-Gemser, 2012). In this respect, it is obviously also important to verify whether adopting self-control in soccer training programs indeed can effectively increase penalty kicking performance in competitive environments.

Future research may also want to more forcefully instruct penalty takers on the strategy to be adopted (i.e., goalkeeper-independent), as well as consider the interaction between goal achievement (intended vs. reached location) and degree of autonomy control of actions. Finally, as is often the case in studies that involve elite players, the present study is low in the number of participants. Although we had the opportunity to include more, but lower skilled participants, we decided against this in order to ensure the high technical ability of participants. Hence, it is important not to overgeneralize the current findings to other groups.

## Conclusion

This is the first experimental evidence that *actual* task control can influence coping with psychological pressure in soccer penalty kicking. Reduced degree of task control in the soccer penalty kicks can enhance the debilitating effects of high pressure as performance was shown to deteriorate when strictly following instruction of others about the aiming location. In addition, a reduced degree of autonomy also was found to result in more rushed behaviors in penalty kickers, irrespective of pressure. Finally, a coach may be inclined to forcefully instruct the players about the distribution of the penalty kicks, especially in a penalty shoot-out. However, such instructions may reduce the degree of task control and, consequently, hinder rather than improve performance, especially in stressful situations. Therefore, and based on the current observations, it seems more advisable to leave to the penalty takers themselves to choose the aiming location.

## Ethics Statement

This study was carried out in accordance with the recommendations of Comité de Ética de la UPM with written informed consent from all subjects. All subjects gave written informed consent in accordance with the Declaration of Helsinki. The protocol was approved by the Comité de Ética de la UPM.

## Author Contributions

JN and JA contributed to the design of the study and data collection. JN performed data analysis. JK led the writing of the paper, and CA and JN significantly contributed to writing process.

### Conflict of Interest Statement

The authors declare that the research was conducted in the absence of any commercial or financial relationships that could be construed as a potential conflict of interest.

## Footnotes

^1^During externally-directed condition, three kicks were intended towards the upper areas of the goal (areas 2, 3 and 4, Figure 1, left) and two kicks towards the lower areas (1 and 5). Taking the midpoints of the upper (1.83 m) and lower areas (0.61 m), the weighted average of the instructed height is 1.34 m.
